# Abdominal pseudohernia in a child after surgical correction of congenital scoliosis: case report

**DOI:** 10.3389/fped.2023.1211184

**Published:** 2024-01-11

**Authors:** Nurbek Nadirov, Sergey Vissarionov, Nikita Khusainov, Alexandra Filippova, Vitaliy Sazonov

**Affiliations:** ^1^ Department of Orthopedics, Mother and Child Health Center, University Medical Center, Astana, Kazakhstan; ^2^ Department of Spine Pathology and Neurosurgery, G. Turner National Medical Research Center for Children’s Orthopedics and Trauma Surgery of the Ministry of Health of the Russian Federation, Saint Petersburg, Russia; ^3^ Department of Surgery, School of Medicine, Nazarbayev University, Astana, Kazakhstan

**Keywords:** pseudohernia, congenital scoliosis, children, complications, hernia of the abdominal wall, case report

## Abstract

Abdominal wall relaxation is a rare complication of various surgical procedures or diseases, when the intercostal or upper lumbar nerves are affected, and the innervation of the muscles of the abdominal wall is damaged. The result is a mass that can visually mimic a ventral hernia. We present a clinical case and the results of a literature review on this topic.

**Clinical case:**

The 2 years 5 months patient diagnosed with a congenital deformity of the spine (posterolateral hemivertebra) underwent extraction of the hemivertebra from the retroperitoneal approach. In the postoperative period, a pseudohernial protrusion of the anterior abdominal wall was observed. 4.5 months later the protrusion resolved spontaneously.

**Discussion:**

Abdominal wall relaxation is studied worldwide and is presented primarily as clinical case reports, mainly in older patients with neurological diseases. Single cases of this pathology are described among children. The Th10-Th12 roots are most often affected. Possible manifestations include: bloating and abdominal pain, pseudo-obstruction of the small and/or large intestine, and constipation. In the described case, only unilateral bloating at rest was observed, which increased with crying and strain. The natural course and prognosis of this diagnosis are usually favorable—the recovery period, according to the literature, takes an average of 4–5 months, which also coincided with our case.

**Conclusion:**

Pseudohernias are a rare complication and may can occur during correction of spinal deformities in children. This condition is a transient disorder of the anterior abdominal wall muscles, the cause of which may be neuropathy caused by infection, metabolic disorders, or mechanical damage. The main principles of treatment of this condition include active observation and symptomatic therapy. The prognosis is usually favorable.

## 1 Introduction

A hernia of the anterior abdominal wall occurs when an organ or a part of it protrudes through a natural opening (such as the umbilical ring, the inguinal or femoral canal) or a defect in the abdominal wall caused by injury or previous surgery (1). Damage to the intercostal or upper lumbar nerves, which innervate the muscles of the abdominal wall, can externally mimic a ventral hernia. However, this type of hernia differs from a true hernia in that there is no weakening of the abdominal wall due to a defect in the muscles and fascial layers. This type of hernia is rare due to the cross-innervation of the abdominal wall developed.

Information on abdominal wall relaxation is presented primarily in the form of case reports and is presented primarily in adult patients with neurological diseases or complications. The literature contains 84 publications for the keyword “pseudohernia,” with more than half of the articles being case reports or case series with small patient samples of pseudohernias caused by herpes zoster infection.

Based on the literature review, it is evident that pseudohernia is a rare complication that occurs due to various surgical interventions or diseases associated with neuropathy or denervation. These complications include infectious and endocrinological diseases [such as herpes zoster (2–4), poliomyelitis (5, 6), and diabetic neuropathy (7)], surgical interventions on the spine (1, 8), and various surgical approaches performed during chest and abdominal surgeries (9–11).

Loewe first described pseudohernia of the anterior abdominal wall in 1936 after injecting a local anesthetic into the abdominal muscles of guinea pigs (12). He noted that the induced relaxation of the abdominal wall occurred without any damage to the musculature of the abdominal wall and called the resulting phenomenon “pseudohernia.” Loewe suggested that the occurrence of pseudohernia is associated with a defect in sensory neurons rather than motor neurons, leading to local relaxation as a result of the interruption of the reflex arc that provides muscle tone to the abdominal wall. Later, damage to motor neurons was described in the pathogenesis of pseudohernias in patients with herpes infection (9–11).

In addition to being a rare complication, there is no consensus among the authors on the optimal treatment of this condition. The literature highlights two fundamentally different approaches to the treatment of pseudohernias: active surveillance with or without symptomatic therapy (13, 14) and surgical repair of pseudohernia (15–17).

Few reports are available about the appearance of pseudohernias of the anterior abdominal wall in children. Therefore, we present a clinical case and the results of a literature review on this topic.

## 2 Case report

At the National Research Center for Pediatric Traumatology and Orthopedics, a 2-year old girl with a diagnosis of “Multiple congenital malformations of the spine” was admitted for planned surgical treatment. The diagnosis was made when she was 3 months old and the main complaint of her mother was the presence of spinal deformity. The conventional therapy, however, could not achieve stability or slowing of progression. Upon admission, the child was able to walk independently without any lameness, but an imbalance of the trunk was observed. Specifically, her right shoulder girdle and shoulder blade were located higher than those of her left shoulder, and the chest was not deformed. Asymmetric waist triangles (*D* < *S*) and a curved spine axis to the right were also observed in the thoracic region. However, the range of motion in the cervical, thoracic, and lumbar spines was not limited and the movements were painless. Additionally, there were no orthopedic pathologies in the upper and lower extremities and no neurological or pelvic organ disorders were detected.

**Figure 1:**
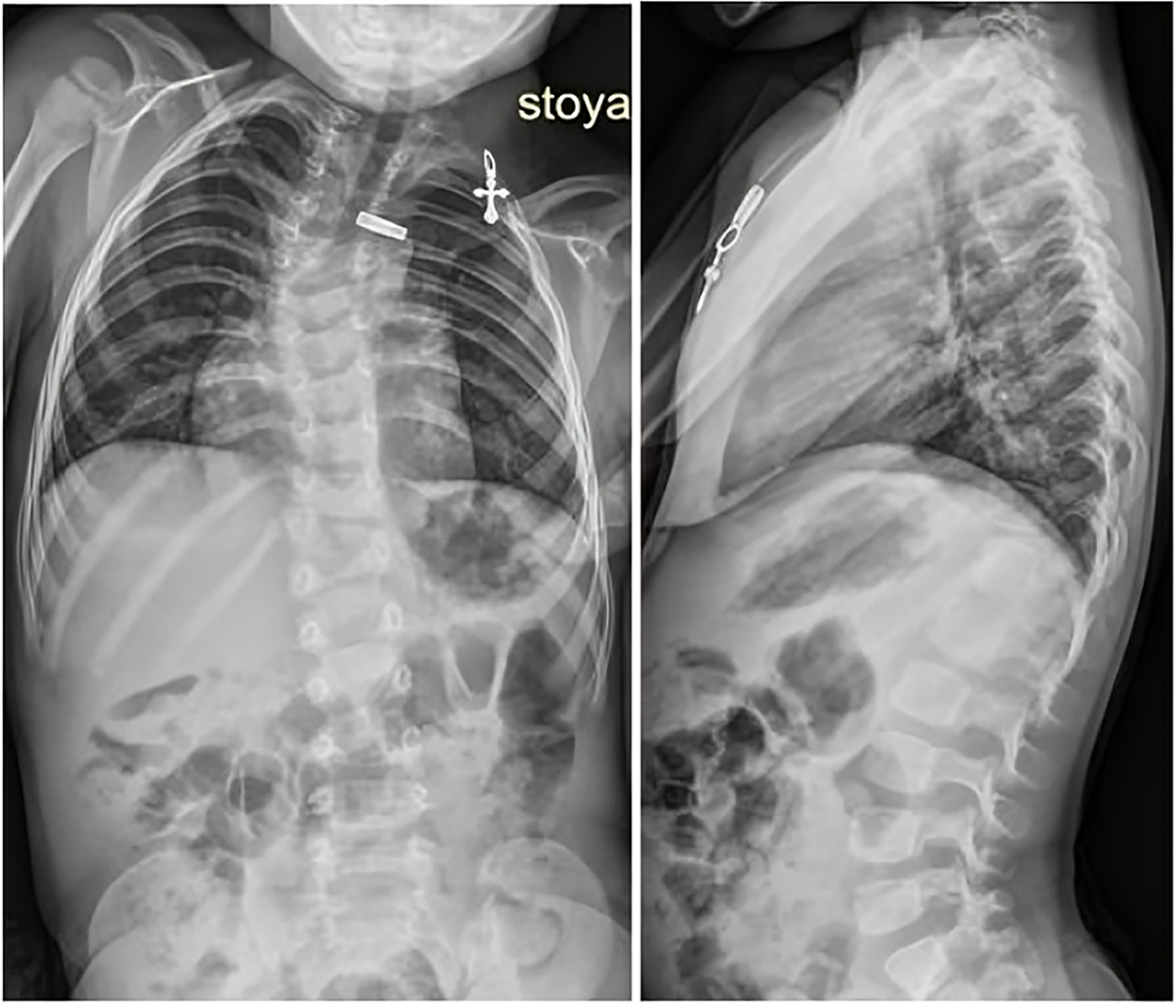
Patient spondylograms, frontal and lateral planes.

The child underwent clinical, laboratory, functional, neurological, and radiological examinations, including multislice computer tomography (MSCT) of the cervical, thoracic, and lumbar spine. The results of the examinations showed multiple congenital spine malformations resulting from impaired formation, fusion, and segmentation of the thoracic vertebrae. Spondylograms and MSCT of the spine revealed a deviation of the thoracic spine to the right due to anomalies in the shape and structure of the thoracic vertebrae. Additionally, an indistinctly differentiated block of the bodies and arches of the vertebrae of the upper and middle thoracic spine was observed. The degree of the right-sided scoliotic curve Th1-Th8 was 35 degrees by Cobb. At the level of the thoracolumbar junction, a right-sided posterolateral hemivertebra Th13 was visualized, forming a local kyphoscoliotic deformity with a scoliotic component of 23 degrees by Cobb and a local kyphosis of the thoracolumbar junction of 33 degrees by Cobb (Figure 1).

Given the results of the examinations and the presence of multiple spine malformations, a multistage surgical treatment was recommended. In this case, risk of developing permanent mobility disability, impaired trunk posture and sitting ability, and restrictive ventilatory impairment due to unstable chest or severe thoracic deformities were also considered. The first step was the extirpation of the posterolateral hemivertebra Th13 (D) using a combined approach. The patient was placed on her left side and anterolateral and extrapleural access was performed on the right side with resection of the 12th rib. The body of the Th13 hemivertebra was extirpated along with the adjacent intervertebral discs. Then, the hemiarch of the Th13 (D) hemivertebra was removed from the dorsal approach and transpedicular supporting elements were installed in the vertebral bodies Th12, Th14 and L1 on both sides to correct the deformity. Posterior and ventral fusion with autologous bone was performed, and a peridural catheter was placed intraoperatively from the dorsal access for anesthesia in the early postoperative period. The wounds were closed. One of the main goals was to allow further growth of the spine, especially the thoracic spine, leading to increased lung volume.

**Figure 2:**
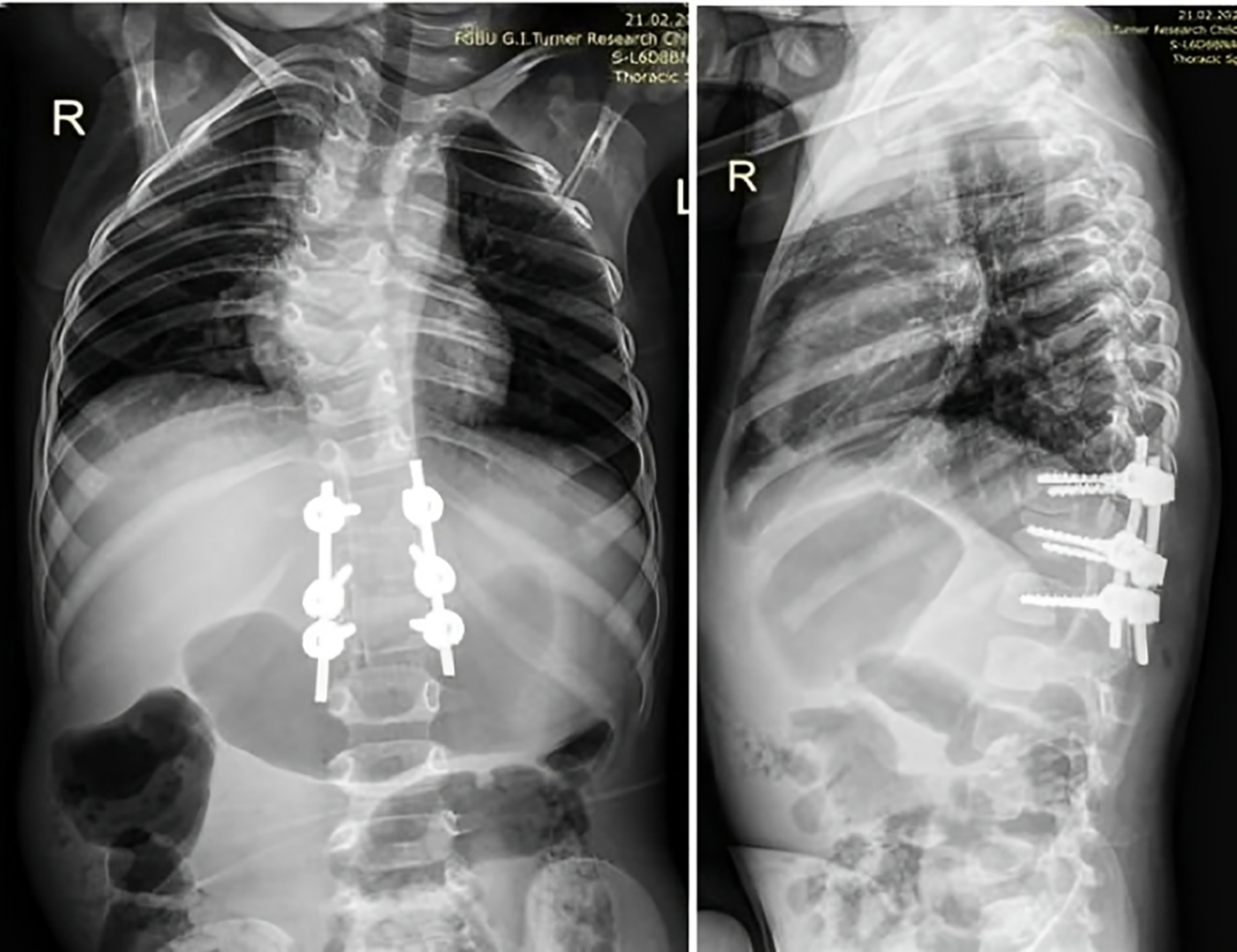
Patient spondylograms, frontal and lateral planes, 5th day post-op.

After completion of surgery and extubation of the child on the operating table, doctors noticed a protrusion on the right side of the anterior abdominal wall. Initially, it was interpreted as relaxation of the abdominal muscles due to the introduction of anesthesia into the peridural space. In the first three days after the operation, the child was kept in the intensive care unit, where a unilateral abdominal protrusion was observed at rest and an increase was observed when the patient cried or strained. The peridural catheter was removed when the child was transferred from the ICU. On the fourth day after surgery, the girl was made to stand, and on the fifth day spondylograms were performed to monitor the correction of the local congenital deformity and the position of the metal structure. The results showed complete correction of the deformity, with the metal structure correctly and stably placed (Figure 2). The muscles of the anterior abdominal wall were found to be intact and there was no presence of free fluid or air in the abdominal cavity, according to MSCT.

**Figure 3:**
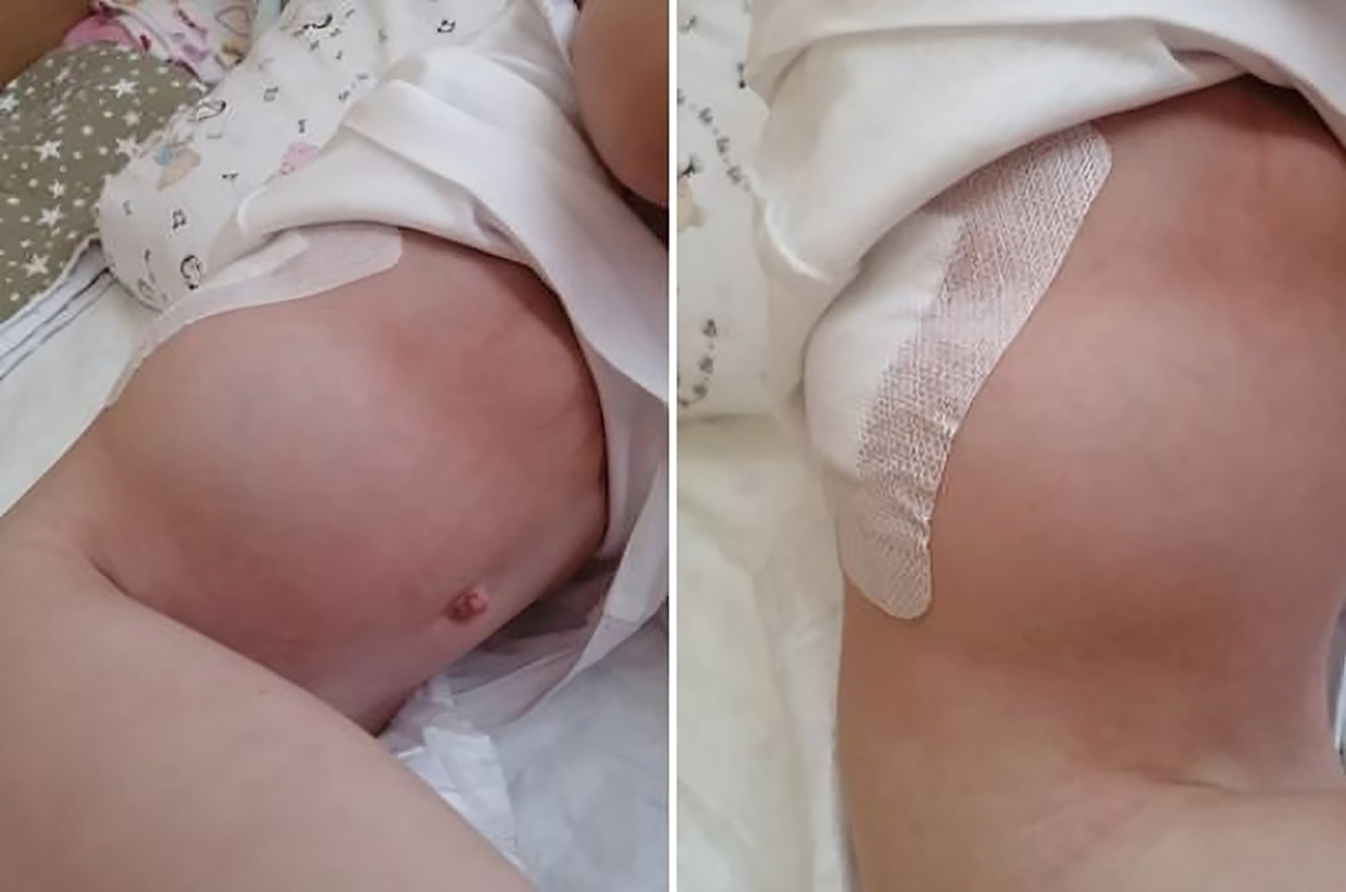
Pseudohernia in a patient in a postoperative period.

The child received perioperative antibiotic prophylaxis, symptomatic therapy, and physical therapy. Surgical wound dressings were changed every three days and wounds healed through primary closure. On the seventh day after surgery, the child was provided with a rigid corrective corset and discharged from the hospital on the 14th day after surgical treatment. Although the pseudohernia of the anterior abdominal wall persisted throughout the hospital stay, it did not cause any complaints from the child (Figure 3).

At the 3-month follow-up examination, the pseudohernial protrusion remained the same size. However, six-month later, the child did not have a pseudohernial protrusion, and according to mother, the spontaneous restoration of the tone of the abdominal wall occurred 4.5 months after surgical intervention.

The protrusion was believed to be a consequence of intraoperative denervation during the lateral transthoracic approach, including resection of the 12th rib and damage to the intercostal nerve due to prolonged use of the wound retractor, or ligation of the neurovascular bundle during wound closure. Neuropathy symptoms included weakness of the abdominal muscles, which manifested as pseudohernia of the anterior abdominal wall.

## 3 Discussion

As mentioned above, pseudohernias are most commonly observed in adult patients as a complication of herpes zoster infection, specifically segmental herpes zoster abdominal paresis. These hernias are the result of damage to the branches of the Th8-L2 spinal nerve. Two extensive reviews have analyzed the incidence, causes, age range of patients, and clinical symptoms of pseudohernias (2, 18). In 88.9% of patients, a herpes zoster skin rash precedes the onset of pseudohernia, typically at the level of the Th11 segment. The complication affects the elderly and immunocompromised patients, mainly men. The average recovery period is 4–5 months, with spontaneous restoration of the tone of the abdominal wall occurring in our patient after 4.5 months following surgery. Pseudohernias can manifest along with other clinical symptoms, including bloating and abdominal pain, pseudo-obstruction of the small or large intestine, and constipation. In our patient, we observed unilateral bloating at rest, which increased with crying and straining. No other clinical signs were observed, and no other neurological deficits were observed, in addition to weakness in the anterior abdominal wall muscles and impaired surface sensitivity locally in the denervated area, which corresponded to intercostal nerve damage.

In addition to herpes zoster, pseudohernias can also result from trauma. Examples include “cough-induced hernias” and pseudohernias that occur after rib fractures with intercostal muscle damage (19–21). Other reported cases of pseudohernias include those associated with diabetic neuropathy (6) and poliomyelitis in children (5).

Surgical denervation of the anterior abdominal wall during a surgical approach is another widely discussed cause of pseudohernias. This occurs often in the lateral transabdominal or lateral thoracoabdominal approaches. In their study, Chatterjee et al. noted a weakness of abdominal wall denervation in 34 patients (49%) during lateral nephrectomy (8). Gardner observed similar symptoms in 20% of patients after an abdominal aortic plasty performed by lateral retroperitoneal approach (22). Other authors described the clinical picture of pseudohernia after thoracoscopic removal of a Th9-Th10 meningioma (10).

The etiology of pseudohernias may be associated with infectious diseases, metabolic disorders complicated by neurological symptoms, iatrogenic (intraoperative denervation), or traumatic causes. Imaging methods such as ultrasound, MSCT, and magnetic resonance imaging (MRI) can be used for diagnostic purposes to confirm the neuropathic genesis of pseudohernias. Unlike true hernias, the muscle-fascial layers of the abdominal wall remain intact in pseudohernias (15, 23, 24).

In most cases, surgical treatment is not necessary for pseudohernias. Active surveillance and/or conservative therapies such as bandage, physical therapy, and symptomatic and analgesic therapy are generally sufficient. The choice of conservative treatment depends on the underlying cause of pseudohernia. For example, patients with herpes zoster may require anti-herpetic drug therapy and adequate analgesia to relieve neuropathic pain (7).

Surgical treatment of pseudohernias is generally considered ineffective, as there is no physical defect of the abdominal wall that can be corrected by plastic surgery (5, 6). However, not all authors agree with this statement. Some recommend surgical treatment through anterior abdominal wall plasty with the installation of a mesh prosthesis by an open or endoscopic method. They suggest this method for pseudohernias resulting from surgical or traumatic denervation, as well as in cases of irreversible neuropathic hypotrophy of the muscles of the denervated area (16, 25).

## 4 Conclusion

Pseudohernias are protrusions of the anterior abdominal wall that resemble true hernias but do not involve a muscular or fascial defect. These bulges are caused by neuropathy or neurological deficits resulting from infection, metabolic disorders, or mechanical damage. The primary approach to the management of pseudohernias involves active observation and symptomatic therapy. In most cases, surgical treatment is considered unnecessary.

## Data Availability

The original contributions presented in the study are included in the article/Supplementary Material, further inquiries can be directed to the corresponding author.
